# Therapeutic Drug Monitoring Versus Fixed-Interval Dosing of Dalbavancin in Implant-Associated Spinal Infections: Grand Round/A Case Study

**DOI:** 10.1097/FTD.0000000000001386

**Published:** 2025-09-19

**Authors:** Jeroen P.A. Houwen, Charlotte S. Hakkers, Valentijn A. Schweitzer, Tim Bognàr, Martha L. Toren-Wielema, Daniël J. Touw, Aurelia H.M. de Vries Schultink

**Affiliations:** * Department of Clinical Pharmacy, University Medical Center Utrecht/Wilhelmina Children's Hospital, Utrecht, the Netherlands;; † Department of Internal Medicine and Infectious Diseases, University Medical Center Utrecht, Georgia, Utrecht, the Netherlands;; ‡ Department of Medical Microbiology, University Medical Center Utrecht, Utrecht, the Netherlands;; § Department of Clinical Pharmacy and Pharmacology, University Medical Center Groningen, Groningen, the Netherlands; and; ¶ Department of Pharmaceutical Analysis, Groningen Research Institute of Pharmacy, University of Groningen, Groningen, the Netherlands.

**Keywords:** implant-associated spinal infection, dalbavancin, therapeutic drug monitoring, antimicrobial therapy

## Abstract

**Background:**

Implant-associated spinal infections (IASI) pose challenges for outpatient management due to the need for frequent intravenous antibiotic administration. Dalbavancin has a prolonged half-life and is a practical alternative.

**Methods:**

Two cases of IASI were treated with dalbavancin in an outpatient setting over 10–12 weeks. One patient received therapeutic drug monitoring (TDM)-guided dosing, while the other was managed with fixed-interval dosing. Dalbavancin plasma concentrations were measured using liquid chromatography-tandem mass spectrometry, and dosing adjustments were guided by pharmacokinetic modeling.

**Results:**

In the TDM-guided case, three dalbavancin doses were sufficient to maintain therapeutic plasma concentrations (≥8 mg/L), whereas the fixed-interval approach required four doses. Both patients successfully completed therapy without recurrence of the infection during follow-up.

**Conclusions:**

TDM-guided dalbavancin therapy optimized drug exposure and reduced the number of doses compared with fixed-interval dosing, highlighting its potential to optimize treatment. Further research is required to establish standardized therapeutic drug monitoring protocols for the management of IASI.

## CLINICIAN

Case 1: A 58-year-old female patient (102.5 kg, creatinine clearance of 71 mL/min based on a 6-hour urine collection) developed a polymicrobial implant-associated spinal infection (IASI) after Th9-L3 spondylodesis and laminectomy at Th12. Intraoperative cultures revealed *Staphylococcus aureus*, *Proteus mirabilis*, and *Enterococcus faecium*. Surgical debridement was performed before dalbavancin therapy.

Intravenous vancomycin and ceftriaxone were initiated to treat *S. aureus*, *P. mirabilis*, and *E. faecium*. *S. aureus* was susceptible to both agents, *P. mirabilis* to ceftriaxone, and *E. faecium* to vancomycin. After 1 week, ceftriaxone was switched to oral ciprofloxacin.

Vancomycin was giving for 4 weeks. Upon discharge, 73 days of the intended 12-week course remained. Linezolid was unsuitable due to long treatment duration and risk of adverse events.^1^

Could dalbavancin effectively cover the remaining treatment duration?

## TDM CONSULTANT

Dalbavancin, a second-generation lipoglycopeptide, inhibits cell wall synthesis by preventing peptidoglycan cross-linking and causing lysis. Its lipophilic side chains improve membrane binding and prolong half-life. Dalbavancin is effective against Gram-positive pathogens, including methicillin-resistant *S. aureus*, *Streptococcus species*, and *Enterococcus species*.^2^ Dalbavancin has been approved for the treatment of skin and soft tissue infections. It is administered as a two-dose (1000 mg on day 1 and 500 mg on day 8) or a single-dose (1500 mg) regimen.^3^ Several studies support its off-label use for severe Gram-positive infections, including IASI, beyond the approved 1–2 dose regimen for acute bacterial skin and skin structural infections.^4–7^

Dalbavancin's long half-life (147–258 hours) enables infrequent dosing, eliminates the need for elastomeric pumps and intravenous access, and reduces phlebitis, line sepsis, and hospital stay—offering clear outpatient advantages over vancomycin and teicoplanin, which require more frequent administration and monitoring.^2^

## CLINICIAN

The patient was discharged and unable to continue daily intravenous therapy; therefore, vancomycin was switched to dalbavancin to complete the remaining 73 days of the planned 12-week antibiotic course after wound closure. Dalbavancin was administered in an outpatient hospital setting, allowing patients to receive a dalbavancin infusion and return home the same day.

How can we adequately administer optimal dalbavancin with optimal exposure?

## TDM CONSULTANT

The pharmacokinetics (PK)/pharmacodynamics (PD) of extended dalbavancin regimens are well described, with most experience using 1500 mg on days 1 and 8 in patients with normal renal function, followed by PK/PD-guided dosing.^8–10^

The first and second doses are often administered 1 week apart.^10,11^ Subsequent TDM can confirm whether a third dose is necessary, and if so, when it should be administered.

## CLINICIAN

The first 1500 mg of dalbavancin was administered on day 1, and TDM was performed on days 4 and 8. The second 1500 mg dose followed on day 8, with TDM on days 22 and 28.

Should a third 1500-mg dose be administered to maintain adequate plasma concentrations, and if so, when?

## TDM CONSULTANT

The PK/PD parameter associated with dalbavancin efficacy is the area under the concentration–time curve for the free drug concentration at 24 hours, divided by the minimal inhibitory concentration (*ƒ*AUC24/MIC) ratio. A target of 111.1, corresponding to a 2-log bacterial kill, was established for *S. aureus* at an MIC of 0.125 mg/L. This translates to a trough concentration of ≥8 mg/L assuming 93% protein binding, although it was validated only in murine *S. aureus* models and not for *E. faecium*.^12^ The Clinical and Laboratory Standards Institute has defined a tentative susceptibility breakpoint of 0.25 mg/L for *E. faecium*.^13^

We targeted a trough concentration of ≥8 mg/L. At a MIC of 0.25 mg/L, this gives an *ƒ*AUC24/MIC of 53.3, approximating a 1-log kill in *S. aureus*. This was considered sufficient due to prior vancomycin therapy.

**FIGURE 1.**
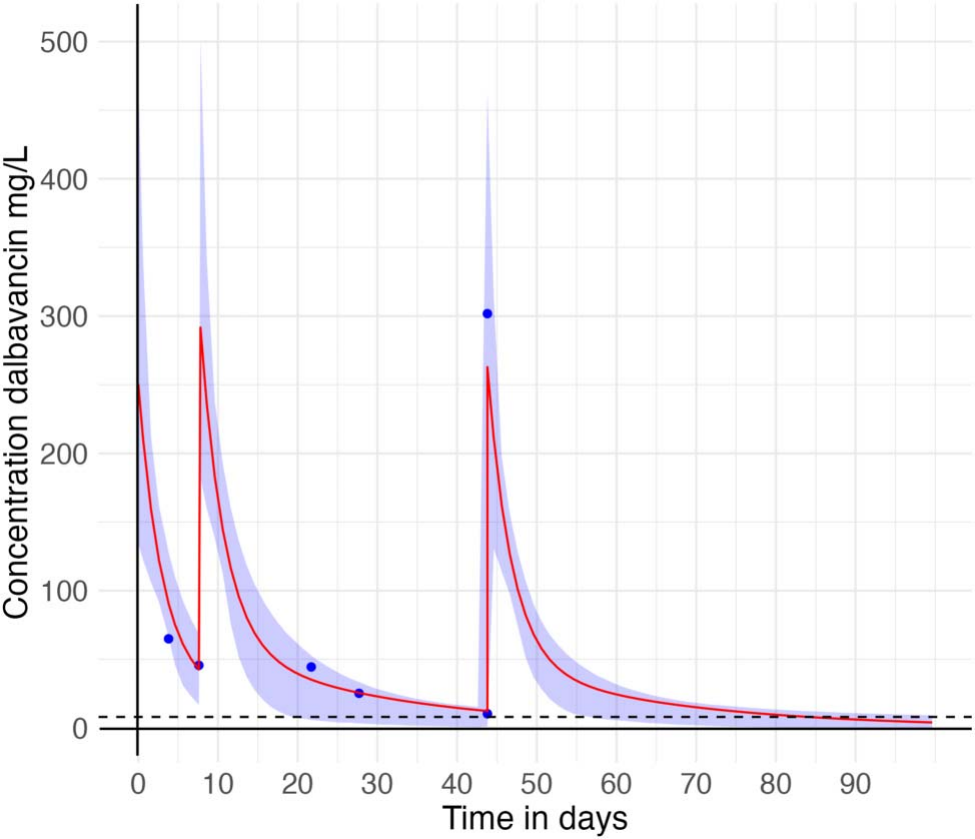
Case 1. Observed (blue dots) and predicted dalbavancin plasma concentrations (red line) over time. Shaded area represents the 95% confidence interval based on uncertainty of the model parameters. Dashed line indicates the threshold of 8 mg/L, reflecting the minimum plasma concentration considered adequate for dalbavancin exposure.

Dalbavancin levels were measured using a validated Liquid chromatography-tandem mass spectrometry assay, according to the European Medicines Agency and International Council for Harmonisation of Technical Requirements for Pharmaceuticals for Human Use guidelines.^14^ Concentrations after 1500 mg doses on days 1 and 9 are shown in Figure 1.

Based on plasma levels and Cojutti's population PK model in MWPharm++, a third 1500-mg dose was administered 35 days after the second dose (day 43). Simulations incorporated patient-specific data to guide personalized dosing and maintain adequate troughs concentration.^9^

## CLINICIAN

A third dose of 1500-mg dalbavancin was administered 36 days after the second dose (day 44 of dalbavancin treatment).

Should a fourth dose be administered to maintain adequate dalbavancin levels for up to 73 days?

## TDM CONSULTANT

The trough concentration before the third dalbavancin infusion was 10.3 mg/L, confirming adequate exposure. After the third dalbavancin infusion, the peak plasma concentration was 301.8 mg/L, and renal function remained stable. The estimated drug exposure was predicted to remain ≥8 mg/L until day 83 (39 days after the last infusion), as shown in Figure 1. This was sufficient for the intended treatment course, which ended on day 73 (29 days after the last infusion). A fourth dose was not required.

## CLINICIAN

Case 2 describes the dalbavancin treatment of a 49-year-old woman (85 kg, eGFR (Chronic Kidney Disease Epidemiology Collaboration) >90 mL/min/1.73 m^2^) with IASI after C3–Th2 spondylodesis and decompression at C4–C7. Intraoperative cultures obtained during repeated surgical irrigations yielded *S. aureus* and *Enterobacter cloacae*. Based on susceptibility, treatments included intravenous vancomycin, oral rifampin, and ciprofloxacin. The spinal implants were then retained. Surgical irrigation was performed before vancomycin initiation. Vancomycin was hospital-administered but was switched to dalbavancin upon discharge because of outpatient challenges.

Dalbavancin was administered in 4 off-label 1500-mg doses every 2 weeks without TDM. The total duration of the antimicrobial treatment was 12 weeks. Creatinine levels remained stable.

After the final dalbavancin dose, the patient developed nausea, vomited once, and had a painful, itchy, erythematous, and swollen face. Symptoms resolved within 2.5 hours after 1-mg oral clemastine (antihistamine) and was discharged.

Could TDM have prevented the adverse reaction and optimized the therapy for dalbavancin?

## TDM CONSULTANT

**FIGURE 2.**
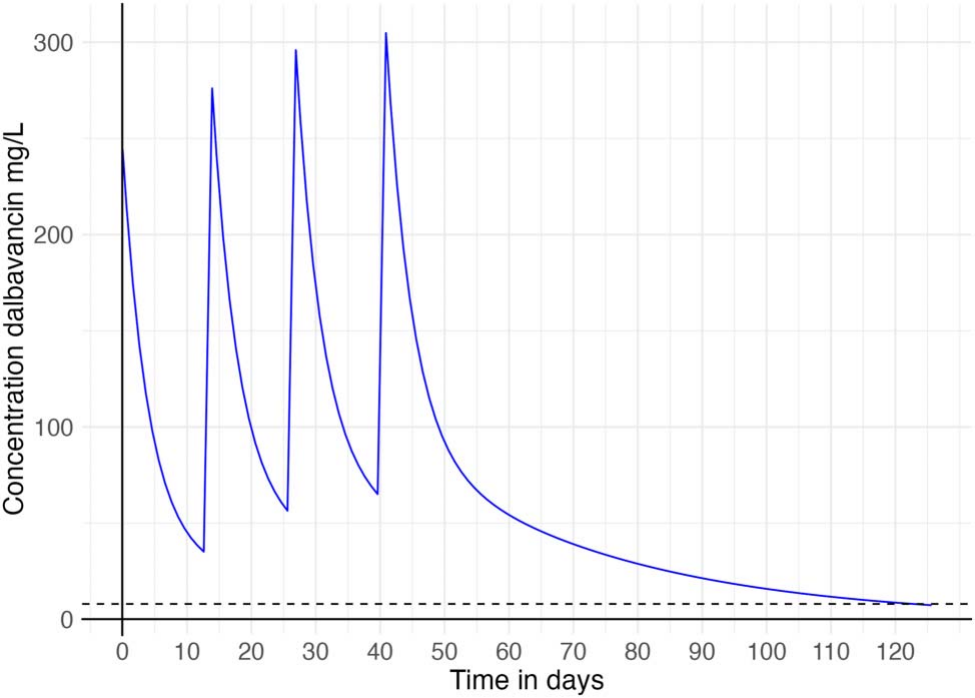
Case 2: Predicted dalbavancin concentrations over time. Dashed line represents the threshold of 8 mg/L, indicating the minimum plasma concentration required for adequate dalbavancin exposure.

MWPharm++ simulations revealed higher peak levels in Figure 2 compared with Figure 1, and higher cumulative trough concentrations due to slower interdose decline, despite preserved renal function.^9^ Although this suggests possible overexposure, no levels were measured, and toxicity thresholds are unknown. The observed infusion reaction may have been related to these levels but could also be coincidental.

TDM could have optimized third-dose timing, reduced costs, and enabled a three-dose course (Fig. 1), thereby highlighting its role in maximizing efficacy and minimizing unnecessary drug exposure.

## CLINICIAN

Both patients completed treatment without infection recurrence up to 8 months after therapy. The second patient had a moderate reaction, possibly due to the infusion. However, blood, renal, and liver tests remained normal.

## CONCLUSION

Two patients with IASI were treated with dalbavancin. Case 1 demonstrates successful TDM-guided dosing to maintain plasma concentrations above the therapeutic threshold of 8 mg/L andreducing the number of doses required. In contrast, case 2 followed a fixed-interval dosing regimen without TDM. TDM-guided dosing enabled accurate target attainment, as proposed by Cattaneo et al.^15^

A possible TDM approach involves measuring trough and peak concentration (30 minutes after infusion) around the second dose to guide third-dose timing. If a third dose is given, follow-up levels can confirm whether concentrations will remain ≥8 mg/L and if a fourth dose is needed.

TDM-guided dalbavancin dosing is an effective outpatient strategy for patients who cannot receive intravenous vancomycin or teicoplanin, reducing the need for prolonged hospital admission. Further research is necessary to establish standardized TDM protocols for dalbavancin to optimize target attainment and enable less frequent dosing. A multidisciplinary approach involving clinicians and TDM consultants is recommended to optimize dalbavancin therapy.

## References

[1] Laarhuis SRE Kerskes CHM Nijziel MR . Linezolid-induced thrombocytopenia in patients with renal impairment: a case series, review and dose advice. Drugs R D. 2024;24:109–115. 38480595 10.1007/s40268-024-00458-6 PMC11035510

[2] Zhanel GG Calic D Schweizer F . New lipoglycopeptides a comparative review of dalbavancin, oritavancin and telavancin. Drugs. 2010;70:859–886. 20426497 10.2165/11534440-000000000-00000

[3] Leuthner KD Yuen A Mao Y . Dalbavancin (BI-387) for the treatment of complicated skin and skin structure infection. Expert Rev Anti Infect Ther. 2015;13:149–159. 25578881 10.1586/14787210.2015.995633

[4] Ciccullo A Giuliano G Segala FV . Dalbavancin as a second-line treatment in methicillin-resistant Staphylococcus aureus prosthetic vascular graft infection. Infection. 2020;48:309–310. 31784895 10.1007/s15010-019-01379-2

[5] Boucher A Pradier M Lafondesmurs B . Dalbavancin as salvage therapy in difficult-to-treat patients for diabetes-related foot osteomyelitis. Infect Dis Now. 2024;54:104835. 37972818 10.1016/j.idnow.2023.104835

[6] Suárez M Pérez-Landeiro A Sanjurjo A . Comparison of dalbavancin with standard of care in the management of infective endocarditis: efficacy, safety, and cost analysis. Int J Infect Dis. 2024;138:41–45. 37931892 10.1016/j.ijid.2023.11.003

[7] Ruiz-Sancho A Núñez-Núñez M Castelo-Corral L . Dalbavancin as suppressive antibiotic therapy in patients with prosthetic infections: efficacy and safety. Front Pharmacol. 2023;14:1185602. 37448966 10.3389/fphar.2023.1185602 PMC10337584

[8] Cojutti PG Rinaldi M Gatti M . Usefulness of therapeutic drug monitoring in estimating the duration of dalbavancin optimal target attainment in staphylococcal osteoarticular infections: a proof-of-concept. Int J Antimicrob Agents. 2021;58:106445. 34614441 10.1016/j.ijantimicag.2021.106445

[9] Cojutti PG Tedeschi S Gatti M . Population pharmacokinetic and pharmacodynamic analysis of dalbavancin for long-term treatment of subacute and/or chronic infectious diseases: the major role of therapeutic drug monitoring. Antibiotics (Basel). 2022;11:996. 35892386 10.3390/antibiotics11080996 PMC9331863

[10] Senneville E Cuervo G Gregoire M . Expert opinion on dose regimen and therapeutic drug monitoring for long-term use of dalbavancin: expert review panel. Int J Antimicrob Agents. 2023;62:106960. 37633424 10.1016/j.ijantimicag.2023.106960

[11] Rappo U Puttagunta S Shevchenko V . Dalbavancin for the treatment of osteomyelitis in adult patients: a randomized clinical trial of efficacy and safety. Open Forum Infect Dis. 2019;6:ofy331. 30648126 10.1093/ofid/ofy331 PMC6326511

[12] Lepak A Marchillo K VanHecker J . Impact of glycopeptide resistance in Staphylococcus aureus on the dalbavancin in vivo pharmacodynamic target. Antimicrob Agents Chemother. 2015;59:7833–7836. 26392492 10.1128/AAC.01717-15 PMC4649188

[13] Weber RE Fleige C Layer F . Determination of a tentative epidemiological cut-off value (ECOFF) for dalbavancin and Enterococcus faecium. Antibiotics (Basel). 2021;10:915. 34438965 10.3390/antibiotics10080915 PMC8388697

[14] European medicines agency. Guideline on bioanalytical method validation; 2011. https://www.ema.europa.eu/en/documents/scientific-guideline/guideline-bioanalytical-method-validation_en.pdf. Accessed June 24, 2025. 10.4155/bio.12.44 22533559

[15] Cattaneo D Fusi M Galli L . Proactive therapeutic monitoring of dalbavancin concentrations in the long-term management of chronic osteoarticular/periprosthetic joint infections. Antimicrob Agents Chemother. 2024;68:e0002324. 38385700 10.1128/aac.00023-24 PMC10989011

